# Exploring the Mechanisms of EDCs-Induced Metabolic Disorders in Humans Using Network Toxicology and Molecular Docking

**DOI:** 10.1016/j.namjnl.2025.100041

**Published:** 2025-08-07

**Authors:** Min Zhao, Yong Niu, Qian Huang, Wenhua Li

**Affiliations:** a School of Medicine, Lijiang University of Culture and Tourism, Lijiang, Yunnan, 674100, China; b School of Medicine, Xizang Minzu University, Xianyang Shaanxi 712082, China

**Keywords:** Endocrine-disrupting chemicals, Network toxicology, Molecular docking, Metabolic diseases, Non-alcoholic fatty liver disease

## Abstract

**Objective:**

This study aims to investigate the potential mechanisms by which EDCs, recognized as emerging pollutants, induce metabolic disorders leading to metabolic diseases in humans.

**Methods:**

Network toxicology and molecular docking techniques were employed to elucidate the molecular mechanisms underlying EDCs-induced pathogenesis of the six diseases. Potential targets associated with EDCs and these diseases were identified using databases such as PubChem, ChEMBL, Super-PRED, GeneCards, OMIM, and TTD. STRING analysis and Cytoscape software were further utilized to determine core targets most significantly linked to these metabolic disorders. GO and KEGG pathway enrichment analyses were performed on the core targets using the DAVID database. Finally, molecular docking was conducted to validate the binding affinities between EDCs and core target proteins.

**Results:**

EDCs may potentially induce metabolic disorders by modulating cellular expression, influencing apoptosis and proliferation, and regulating related signaling pathways. Notably, a close interrelationship was observed among lipid metabolism disorders and atherosclerosis, Alzheimer’s disease, type 2 diabetes mellitus, osteoporosis, hyperuricemia, and non-alcoholic fatty liver disease.

**Conclusion:**

This study provides novel insights into the mechanisms through which EDCs induce metabolic diseases in humans and highlights correlations among distinct disorders, thereby establishing a theoretical foundation for disease prevention and therapeutic strategies.

## 1 Introduction

Endocrine-disrupting chemicals (EDCs), also termed environmental hormones, represent a class of "emerging micropollutants". These compounds are extensively utilized in daily life applications including plastic products (e.g., bisphenol A in food packaging), pesticides (e.g., organochlorine compounds), personal care products (phthalate-based plasticizers), and industrial flame retardants (Minye et al., 2024). However, these chemicals enter human bodies through food chains, air, water systems, and consumer goods. Through interference with hormone synthesis, secretion, receptor binding, or metabolic pathways, chronic low-dose exposure induces cumulative effects, triggering health risks such as reproductive dysfunction, metabolic disorders, and carcinogenesis (Yu and Ying, 2025; Xiao et al., 2023).Recent years have witnessed EDCs being classified as priority organic pollutant groups, driving intensified research focus. Significant advancements have been made in understanding their reproductive toxicity. For instance, phthalates (e.g., DEHP) induce ferroptosis-mediated testicular and Leydig cell damage in mice while suppressing testosterone levels (Fengqiong et al., 2024);Bisphenol A mimics estrogenic activity, elevating risks of breast cancer and premature ovarian insufficiency in females (Dumitrascu et al., 2020). Concurrently, EDCs' metabolic interference has garnered substantial attention. Epidemiological studies demonstrate strong associations between per- and polyfluoroalkyl substances (PFAS) exposure and obesity, insulin resistance, and type 2 diabetes, mediated through aberrant activation of peroxisome proliferator-activated receptor signaling pathways (Ji et al., 2023). Animal experiments further confirm that low-dose EDC exposure induces hepatic lipid metabolism dysregulation, accelerating non-alcoholic fatty liver disease progression (Dongxiao, 2025; Peiyun et al., 2023).

The ecological ramifications of EDCs are equally severe. Their environmental persistence and bioaccumulation lead to sex ratio imbalances and reproductive impairments in aquatic organisms (e.g., fish and amphibians), threatening biodiversity[2, 9]Although regulatory frameworks like the EU's REACH legislation have restricted certain EDCs, developing nations face pronounced contamination challenges due to regulatory lags (Guil-Oumrait et al., 2024).For example, surface waters and sediments in China's Pearl, Yangtze, and Yellow River basins exhibit significantly elevated EDC concentrations, with steroid hormones (e.g., 17α-ethynylestradiol) posing particularly high ecological risks (Liang et al., 2016).Nevertheless, the mechanisms underlying EDCs' potential health risks in human diseases remain incompletely understood, with current research in this domain remaining limited. Building upon these findings, we selected seven EDCs prevalent in natural water bodies for metabolic disease analysis. Detailed information including molecular formulas, molecular weights, and SMILES structures of these EDCs is provided in Table 1. And the concentrations of seven pollutants in part of water bodies in Table 2.

**Table 1:** Molecular formula, molecular weight and SMILES structure of 7 EDCs.

Name	molecular formulas	molecular weights (g/mol)	SMILES structures
DDT	C14H9Cl5	354.5 g/mol	C1=CC(=CC=C1C(C2=CC=*C*(*C* = C2)Cl)C(Cl)(Cl)Cl)Cl
Estrone	C18H22O2	270.4 g/mol	C[C@]12CC[C@H]3[C@H]([C@@H]1CCC2=*O*)CCC4=C3C=CC(=C4)O
Dibutyl phthalate	C16H22O4	278.34 g/mol	CCCCOC(=*O*)C1=CC=CC=C1C(=*O*)OCCCC
Triclosan	C12H7Cl3O2	289.5 g/mol	C1=CC(=*C*(*C* = C1Cl)O)OC2=*C*(*C* = *C*(*C* = C2)Cl)Cl
Bisphenol A	C15H16O2	228.29 g/mol	CC(C)(C1=CC=*C*(*C* = C1)O)C2=CC=*C*(*C* = C2)O
Bisphenol F	C13H12O2	200.23 g/mol	C1=CC(=CC=C1CC2=CC=*C*(*C* = C2)O)O
4,4′-Sulfonyldiphenol	C12H10O4S	250.27 g/mol	C1=CC(=CC=C1O)S(=*O*)(=*O*)C2=CC=*C*(*C* = C2)O

**Table 2:** The concentrations of 7 pollutants in part of water bodies.

pollutant	Water body	Concentration (ng/L)	references
DDT	The waters of Xinghua Bay in Fujian	0.77–8.11 ng/L	
	Dagu River in Tianjin	0.33–1.76 ng/L	
	suburban rivers in Beijing	3.51–5.57 ng/L	
	Guanting Reservoir Yongding River in Beijing	1.69–15.20 ng/L	
	Tonghui River in Beijing	14.89–415.10 ng/L	
	Minjiang River in Fujian	10.26–53.57 ng/L	
	Daliao River	0.20–0.90 ng/L	
	Qiantang River	1.64–3.41 ng/L	
	Liaohe River	ND-1.45 ng/L	
	Haihe River	0.023–0.14 ng/L	
	Huangpu River	19.56–26.91 ng/L	
	Jiulong Rive	0.03–9.73 ng/L	
	Taicha Lake	0.31–14.10 ng/L	
	West Lake in Hangzhou	ND-1.86 ng/L	
Estrone	Jiangsu section of the Yangtze River	1.00 ± 1.72 ng/L	
	Liangzi Lake	0.48–0.77 ng/L	
	the Pearl River	4.2–17.2 ng/L	
	main drinking water in main urban area of Kunming	1.98 ng/L	
	lake water in Minnesota	0.05–1.5 ng/L	
Dibutyl phthalate	Guanlan River Basin	ND-5831.70 ng/L	
	Fenhe River	1.0–16.53 μg/L	
	The middle and lower reaches of the Yellow River	ND-26 μg/L	
	the lower reaches of the second Songhua River	ND-5616.80 μg/L	
	the Chaohu Lake in Anhui Province	1368–12.95 μg/L	
	the Wei River basin	0.365–40.68 μg/L	
	the Hai River,	0.041 μg/L	
	the Wuhan section of the Yangtze River	0.156–1.71 μg/L	
	the surface water of Nanchang City	1390 μg/L	
	the Kunming Lake in Beijing	5.43–7.25 μg/L	
	the source water of Hefei	0.41–4.78 μg/L	
	the Beijing Tianjin region basin	0.94–3.6 μg/L	
	the surface water of Guangzhou	1.0–13.5 μg/L	
	the rivers in Taiwan	1.0–44.3 μg/L	
	Italy	2.8–12.19 μg/L	
	the East London Port in South Africa	0.21 μg/L	
	the Klang River in Malaysia	0.8–4.8 μg/L	
	the Velino River in Italy	ND-44.3 μg/L	
	the Dutch Bay	0.07–3.1 μg/L	
Triclosan	139 rivers in 30 states of the United States	<LOQ-2300 ng/L	
	Glat River/Griffin Lake/Zurich River in Switzerland	<0.4–74.0 ng/L	
	lcavcri/vellar/tambaruparani River in India	2–5160 ng/L	
	wutong River in Hong Kong	8.0–83.6 ng/L	
	Lincun River in Hong Kong	1.2–133 ng/L	
	Liao River Basin	2.40–404 ng/L	
	Hai River	ND-34.4 ng/L	
	Yellow River	ND-64.7 ng/L	
	the Pearl River Basin	1.51–478 ng/L	
	Dongjiang River Basin	<LOQ-170 ng/L	
	Jiulong River Basin	<MDL-64.0 ng/L	
Bisphenol A	European waters	<0.03–588,000 ng/L	
	Surface water of the Taihu Lake	27–565 ng/L	
	Songhua River (Harbin)	22–49 ng/L	
	Liao River	5.9–141.0 ng/L	
	Hun River	44–107 ng/L	
	Hai River	ND-106 ng/L	
	Changjiang (Shanghai)	7.1–190 ng/L	
	Yangtze River Estuary (Shanghai)	ND-1.4 ng/L	
	Changjiang River (Chongqing Yichang)	ND-50.1 ng/L	
	Changjiang River (Nanjing)	ND-563 ng/L	
	Suzhou River (Shanghai)	9.2–15.0 ng/L	
	rivers in Taizhou City	ND-1200 ng/L	
	rivers in Wenzhou City	170–2130 ng/L	
	Yongjiang River (Ningbo)	15–1415 ng/L	
	the Pearl River	43–639 ng/L	
	Beijiang River	0.06–0.72 ng/L	
	Liuxi River (Guangzhou)	76–7480 ng/L	
	Taihu Lake	28–560 ng/L	
	Luoma Lake	49–110 ng/L	
	East Lake	ND-37 ng/L	
Bisphenol F	Surface water of the Taihu Lake	ND-1634 ng/L	
	Fuhe	3.09±1.20 ng/L	
	Baiyangdian	1.02±1.23 ng/L	
4,4′-Sulfonyldiphenol	Surface water of the Taihu Lake	4.5–1569 ng/L	
	The Pearl River	1.6–59.8 ng/L	
	Yangtze River	0.18–14.9 ng/L	
	Luoma Lake	ND-94 ng/L	
	Liaohe River	0.22–5.2 ng/L	
	Hunhe River	0.61–46 ng/L	

Metabolic diseases are a group of chronic disorders characterized by energy metabolism imbalance, primarily including obesity, type 2 diabetes mellitus (T2DM), non-alcoholic fatty liver disease (NAFLD), and atherosclerosis. Recent studies have identified exposure to endocrine-disrupting chemicals (EDCs) as a significant contributor to metabolic disorders. For instance, bisphenol A (BPA) and phthalates promote adipocyte differentiation and lipid accumulation by activating peroxisome proliferator-activated receptor gamma (PPARγ) and glucocorticoid receptors, thereby exacerbating obesity (Schaffert et al., 2022; Xinda et al., 2023). Per- and polyfluoroalkyl substances (PFAS) impair glucose uptake through inhibition of insulin receptor substrate phosphorylation, leading to insulin resistance (Gui et al., 2023). Clinical investigations further demonstrate a dose-response relationship between EDC exposure and metabolic syndrome risk (Schug et al., 2016).Notably, gender-specific differences in metabolic susceptibility to EDCs have been observed: females exhibit higher vulnerability to thyroid dysfunction and obesity due to enhanced estrogen receptor sensitivity (Zhihua et al., 2022).Our KEGG enrichment analysis identified six metabolic disorders: lipid metabolism/atherosclerosis, Alzheimer's disease, T2DM, osteoporosis, hyperuricemia, and NAFLD. Atherosclerosis represents a chronic arterial wall pathology primarily driven by lipid deposition. This process involves accumulation of lipids and extracellular matrix proteins, along with calcification of the intima and media, ultimately causing arterial remodeling and reduced elasticity (Tomiyama et al., 2017; Chen et al., 2016). Dysregulated lipid metabolism serves as both a risk factor and hallmark of atherosclerosis, underscoring its critical role in metabolic regulation (Poznyak et al., 2020). Alzheimer's disease, an age-related neurodegenerative disorder, arises from complex interactions between genetic and environmental factors. Emerging evidence reveals intricate connections between lipid metabolism and key pathogenic mechanisms in AD progression (Yin, 2022).T2DM, characterized by insufficient insulin production or impaired utilization, has been linked to abnormal lipid metabolism in Chinese population-based lipidomic studies (Zhong et al., 2017).Osteoporosis manifests as a systemic skeletal disorder featuring reduced bone mineral density, deteriorated bone microstructure, and increased fracture susceptibility. Lipid metabolism disturbances disrupt bone homeostasis by promoting osteoblast-derived cytokine secretion and stimulating osteoclast differentiation (Zhang et al., 2024). Hyperuricemia, a chronic metabolic condition resulting from purine metabolism dysfunction, induces lipid dysregulation through LPCAT3-mediated suppression of p-STAT3 and activation of SREBP-1c (Liu et al., 2020). NAFLD, a clinicopathological syndrome characterized by excessive hepatic lipid deposition unrelated to alcohol consumption, stems from acquired metabolic stress closely associated with insulin resistance and genetic predisposition. Abnormal lipid metabolism, particularly fatty acid accumulation, arachidonic acid dysregulation, and ceramide overload, directly or indirectly contributes to NAFLD pathogenesis (Pei et al., 2020).

Network toxicology represents an integrated research methodology combining systems biology, computational modeling, and big data analytics to elucidate multi-target toxicity mechanisms of chemical substances. Its core approach involves constructing "compound-gene-pathway-disease" interaction networks to identify critical toxicological nodes and signaling pathways (He et al., 2024; Huang, 2023; Tao et al., 2013). Compared with conventional toxicological methods, network toxicology demonstrates superior efficiency in deciphering synergistic or antagonistic effects of complex pollutant mixtures.

Molecular docking, a computational chemistry-based simulation technique, predicts ligand-biomacromolecule binding conformations and affinity to reveal interaction mechanisms. The core procedure encompasses conformation sampling, energy optimization, and binding free energy calculation, providing valuable insights into potential therapeutic interactions or adverse effects (Sukumaran et al., 2024; Stanzione et al., 2021). When applied in toxicological investigations, this technique enables prediction and clarification of toxin-biomolecule interactions, elucidating toxicity mechanisms and potential hazards these substances may impose on organisms (He et al., 2024).

This study aims to systematically investigate the impact of prevalent endocrine-disrupting chemicals (EDCs) in soil and aquatic environments on metabolic disorders. We focus on elucidating molecular interactions and associated mechanisms between seven EDCs and key proteins involved in metabolic diseases. To this end, we will employ integrated network toxicology and molecular docking approaches. These methodologies will enable comprehensive mapping of compound-target relationships, thereby providing mechanistic insights into how EDCs perturb metabolic pathways and drive disease pathogenesis. Through this multidimensional analysis, we seek to unravel the molecular basis of EDCs-induced health consequences and identify potential intervention points for mitigating their adverse effects.

## 2 Methods

### 2.1 Acquisition of EDCs targets

The seven identified compounds were individually queried in the PubChem database (Kim et al., 2023) (https://pubchem.ncbi.nlm.nih.gov/) to obtain their SMILES identifiers. These identifiers were subsequently submitted to Swiss Target Prediction (http://swisstargetprediction.ch), the ChEMBL database (Gaulton et al., 2017) (https://www.ebi.ac.uk/chembl/), and the Super-PRED database (Deng et al., 2025) (https://prediction.charite.de), with the species filter restricted to “Homo sapiens”. Target names were standardized using the UniProt database (Consortium, 2023) (https://www.uniprot.org). Following the combination and deduplication of prediction results, the final ECDs targets were compiled.

### 2.2 Construction of disease targets

Disease-associated targets were primarily retrieved from the GeneCards database (https://www.genecards.org/), OMIM (https://www.omim.org/), and the Therapeutic Target Database (TTD; https://db.idrblab.net). Keyword searches included "metabolic diseases," "lipid metabolism and atherosclerosis," "Alzheimer's disease," "type 2 diabetes mellitus (T2DM)," "osteoporosis," "hyperuricemia," and "non-alcoholic fatty liver disease (NAFLD)." To ensure high relevance to metabolic disorders and ECDs effects, the GeneCards 'score' threshold was set to the median value, and genes with scores exceeding the median were selected. After merging and deduplicating targets from the three databases, the final disease-specific targets were obtained as follows: metabolic diseases (7011 targets), lipid metabolism and atherosclerosis (4862 targets), Alzheimer’s disease (4989 targets), T2DM (6242 targets), osteoporosis (7264 targets), hyperuricemia (1385 targets), and NAFLD (1952 targets).

### 2.3 Identification of overlapping genes between ECDs targets and disease targets

The overlapping genes between compound targets and disease targets were identified using the Venn diagram tool (https://bioinformatics.psb.ugent.be/webtools/Venn/). The target genes of ECDs and the metabolic disease-related target database were uploaded to the platform, and the intersection results were obtained after submission.

### 2.4 Construction of protein-protein interaction (PPI) network

The PPI network was generated by importing overlapping genes from the Venn diagram into the STRING database (https://cn.string-db.org/). The species was set to “Homo sapiens”, and an interaction score ≥0.4 was applied to construct the PPI network. The STRING results were imported into Cytoscape software for network visualization and topological analysis. CytoHubba plugin with the Degree algorithm was utilized to predict the top hub genes.

### 2.5 GO and KEGG pathway enrichment analysis

Functional enrichment analysis, including Gene Ontology (GO) terms (biological processes, molecular functions, and cellular components) and KEGG pathways, was performed using the DAVID database (https://david.ncifcrf.gov/) with “Homo sapiens” as the species. This analysis aimed to elucidate critical signaling pathways associated with core targets in metabolic diseases. Visualization tools from the Microbioinformatics platform were employed to interpret and graphically present the GO and KEGG results.

### 2.6 Construction of compound-target-pathway network

A compound-target-pathway network was established to visualize the regulatory effects of compounds on key pathways and their interactions with targets. The network was generated using Cytoscape 3.8.2 (http://www.cytoscape.org/).

### 2.7 Molecular docking

Molecular docking was conducted to analyze interactions between compounds and core target proteins. The 3D structures of small-molecule ligands were retrieved from PubChem (https://pubchem.ncbi.nlm.nih.gov), while the tertiary structures of core proteins were predicted using AlphaFold 3 (https://alphafoldserver.com/). Water molecules and native ligands were removed from target proteins using PyMOL, followed by hydrogenation, charge calculation, and nonpolar hydrogen merging in AutoDock Vina 1.5.6. After defining grid box parameters and genetic algorithm settings, docking simulations were performed. Discovery Studio 4.5 and PyMOL 1.7.x were used for result visualization.

## 3 Results

### 3.1 Impact of EDCs on metabolic diseases

**Fig. 1:**
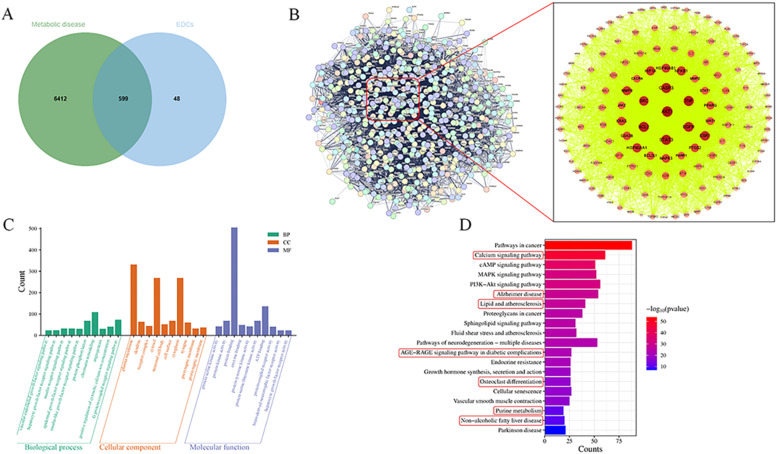
The associations between EDCs and metabolic diseases. (A) The Venn diagram of related targets between metabolic diseases and EDCs. (B) PPI network diagram of core targets. (C) GO enrichment analysis. (D) KEGG enrichment analysis of core targets.

We obtained 647 targets for EDCs from the Swiss Target Prediction website, ChEMBL database, and Super-PRED database. Additionally, we identified 7011 disease-related targets from GeneCards, OMIM database, and TTD database. By integrating these targets, we generated a Venn diagram (Fig. 1A) illustrating the overlap between EDCs and metabolic disease targets. The intersecting region revealed 599 potential targets specifically associated with metabolic diseases induced by EDCs.

Subsequently, we constructed a PPI network for these overlapping genes using the STRING database. The results of this network analysis were imported into Cytoscape version 3.8.2 for further optimization. We employed the Degree algorithm from the CytoHubba plugin to predict core targets; specifically, we selected those with Degree >50 to identify key nodes in the PPI network (Fig. 1B).

We then performed Gene Ontology (GO) and Kyoto Encyclopedia of Genes and Genomes (KEGG) enrichment analyses on the core targets using the DAVID database with “Homo sapiens” as the species setting. This analysis yielded 175 KEGG entries along with findings related to 1155 biological processes (BP), 161 cellular components (CC), and 397 molecular functions (MF). In our GO enrichment results, we highlighted the top ten entries in each category—BP, CC, and MF—and created a corresponding GO enrichment analysis chart (Fig. 1C). For KEGG enrichment analysis, we selected the top twenty entries and illustrated them accordingly in Fig. 1D

As shown in Fig. 1C, BP categories are primarily associated with processes such as protein phosphorylation and chromatin remodeling among others. The CC category is mainly linked to cellular compartments like plasma membrane and neuronal cell body. Meanwhile, MF categories predominantly involve interactions such as protein binding and ATP binding. Fig. 1D displays relevant disease pathways including lipid metabolism disorders leading to atherosclerosis; Alzheimer’s disease; diabetes mellitus; osteoclast differentiation; non-alcoholic fatty liver disease; and purine metabolism disorders—therefore prompting us to conduct an in-depth analysis of six specific diseases: atherosclerosis due to lipid dysregulation; Alzheimer’s disease; type II diabetes mellitus; osteoporosis; hyperuricemia; and non-alcoholic fatty liver disease.

**Fig. 2:**
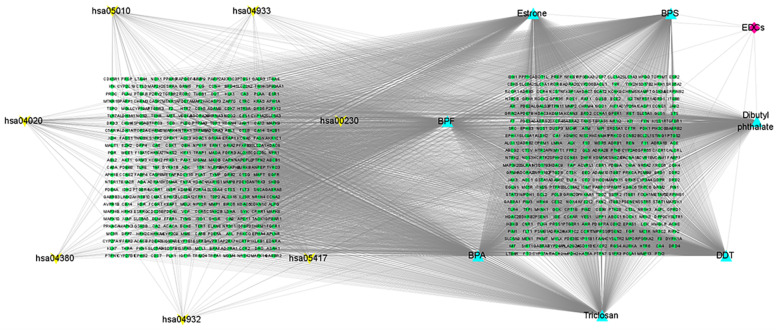
Compound-target-pathway network diagram of EDCs induced metabolic diseases.

In Fig. 2, we visually represented a compound-target-pathway network using Cytoscape software. This network diagram includes seven components of EDCs depicted as light blue diamonds alongside seven pathways indicated by yellow inverted triangles while relevant target genes are denoted by green circles. This visualization effectively illustrates complex relationships among EDCs components across seven metabolic pathways along with their associated target genes.

### 3.2 The impact of EDCs on lipids and atherosclerosis

**Fig. 3:**
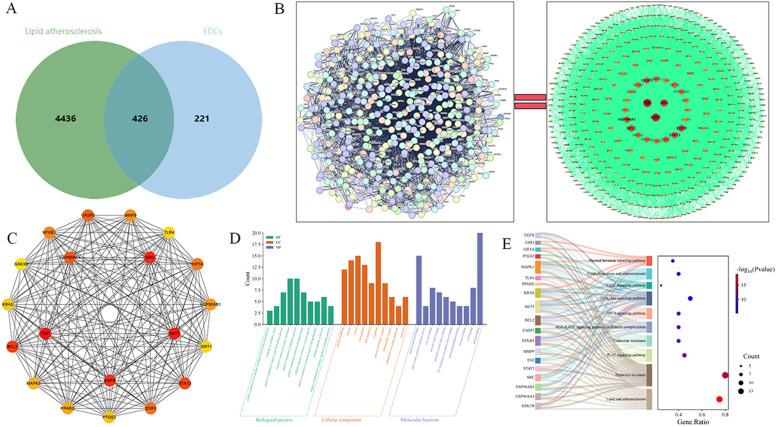
The associations between EDCs and lipid and atherosclerosis. (A) The Venn diagram of related targets between lipid and atherosclerosis and EDCs. (B) PPI network diagram of core targets. (C) Top 20 Hubba gene of core targets.(D) GO enrichment results. (E) The top 10 KEGG enrichment results.

We obtained a total of 4862 genes related to lipids and atherosclerosis from the GeneCards database, OMIM database, and TTD database, along with 647 genes associated with EDCs to construct a Venn diagram (Fig. 3A). This diagram reveals the relationship between EDCs targets and those related to lipids and atherosclerosis, highlighting a set of 466 potential targets specifically associated with EDCs-induced lipid metabolism and atherosclerosis.

Using the STRING database, we constructed a PPI network for these 466 potential targets and imported the results into Cytoscape software for further analysis (Fig. 3B). In Cytoscape, we employed the Degree algorithm from the CytoHubba plugin to classify disease-related targets associated with lipids and atherosclerosis, identifying the top 20 core genes (Fig. 3C).

Subsequently, we performed GO and KEGG enrichment analyses on these 20 core genes using the DAVID database, setting the species as “Homo sapiens”. This generated 132 KEGG entries alongside 250 biological processes (BP), 30 cellular components (CC), and 51 molecular functions (MF). In our GO enrichment results, we highlighted the top ten entries in BP, CC, and MF categories respectively while creating visual representations of these findings in Fig. 3D For KEGG enrichment analysis, we selected the top ten entries for corresponding graphical representation (Fig. 3E). The results indicated that BP categories were primarily linked to negative regulation of apoptotic processes, positive regulation of protein phosphorylation, as well as responses to oxidative stress; CC was mainly associated with cytoplasm, cytosol, and nucleus; whereas MF predominantly involved protein binding, ATP binding,and enzyme binding. Furthermore, KEGG pathway analysis revealed that pathways relevant to EDCs-induced lipid metabolism and atherosclerosis are connected with Lipid Metabolism & Atherosclerosis, Pathways in Cancer, and PI3K-Akt Signaling Pathway among others. These findings provide valuable insights into themolecular mechanisms underlying EDCs-induced lipid metabolismandatherosclerosis.

**Fig. 4:**
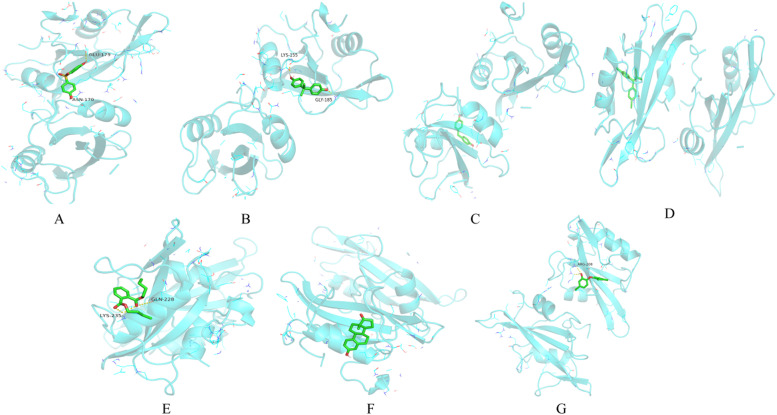
Molecular docking. (A) 4,4 '- Sulfonyldiphenyl docked with SRC; (B) Bisphenol A docking with SRC; (C) Bisphenol F docking with SRC; (D) DDT docking with SRC; (E) Dibutyl phthalate docking with SRC; (F) Estrone docking with SRC; (G) Triclosan docking with SRC.

The molecular docking of SRC with seven different types of EDCs revealed a strong binding affinity between these EDCs and the SRC protein. Fig. 4 visualizes the complexes formed by the seven EDCs and SRC, showcasing their lowest binding energy configurations. The validation results from molecular docking are presented in Table 3.

**Table 3:** Verification results of molecular docking.

targets	Ingredients	Affifinity (kcal/mol)	Distance from best mode	
			rmsd l.b.	rmsd u.b.
SRC	4,4 '- Sulfonyldiphenyl	−5.7	0	0
	Bisphenol A	−5.0	0	0
	Bisphenol F	−4.3	0	0
	DDT	−5.3	0	0
	Dibutyl phthalate	−3.9	0	0
	Estrone	−6.3	0	0
	Triclosan	−5.1	0	0

### 3.3 Impact of EDCs on alzheimer's disease

**Fig. 5:**
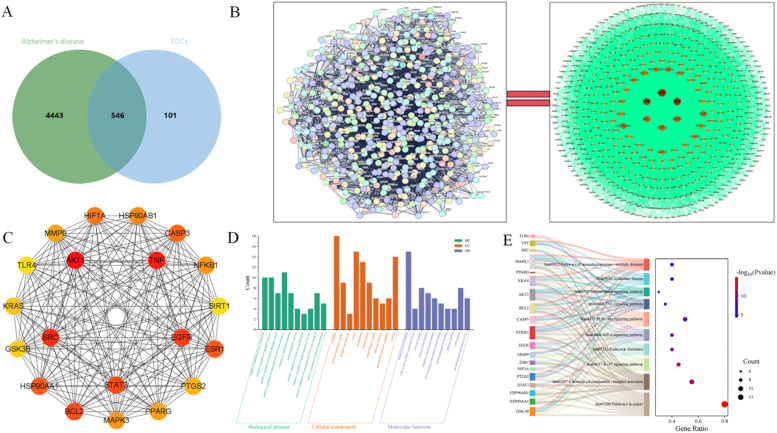
The associations between EDCs and Alzheimer's disease. (A) The Venn diagram of related targets between Alzheimer's disease and EDCs. (B) PPI network diagram of core targets. (C) Top 20 Hubba gene of core targets.(D) GO enrichment results. (E) The top 10 KEGG enrichment results.

We constructed a Venn diagram (Fig. 5A) using data from GeneCards, OMIM, and TTD databases, which included a total of 4989 genes associated with Alzheimer's disease and 647 genes related to EDCs. This diagram illustrates the relationship between the targets of EDCs and those implicated in Alzheimer's disease, highlighting a set of 546 potential targets specifically associated with EDCs-induced Alzheimer’s pathology.

Utilizing the STRING database, we developed a PPI network for these 546 potential targets and imported the results into Cytoscape software for further analysis (Fig. 5B). In Cytoscape, we employed the Degree algorithm via the CytoHubba plugin to classify disease-related targets linked to Alzheimer's disease, identifying the top twenty core genes (Fig. 5C).

Subsequently, we performed GO and KEGG enrichment analyses on these twenty core genes using DAVID database tools; our species was set as “Homo sapiens”. This analysis yielded 159 KEGG entries along with findings across various biological processes (BP), cellular components (CC), and molecular functions (MF)—specifically comprising 250 BP terms, 30 CC terms, and 51 MF terms. Within our GO enrichment results, we highlighted the top ten entries for BP, CC, and MF respectively while creating corresponding visualization charts for GO enrichment analysis (Fig. 5D). For KEGG pathway analysis purposes, we selected ten leading entries to illustrate relevant pathways graphically (Fig. 5E). The outcomes indicated that BP categories were primarily associated with negative regulation of apoptotic processes; positive regulation of transcription by RNA polymerase II; as well as neuron apoptotic processes among others. The CC category predominantly involved cytoplasm; neuronal cell body; nucleus; etc., while MF was mainly concerned with identical protein binding; ATP binding; enzyme binding; etc.. Furthermore, the KEGG pathway analysis suggested that pathways related to neurodegeneration—multiple diseases,pathways in cancer—and PI3K-Akt signaling pathway were significantly connected to EDCs-induced Alzheimer’s disease mechanisms. These findings provide valuable insights into understanding the molecular mechanisms underlying EDCs-induced Alzheimer’s disease.

**Fig. 6:**
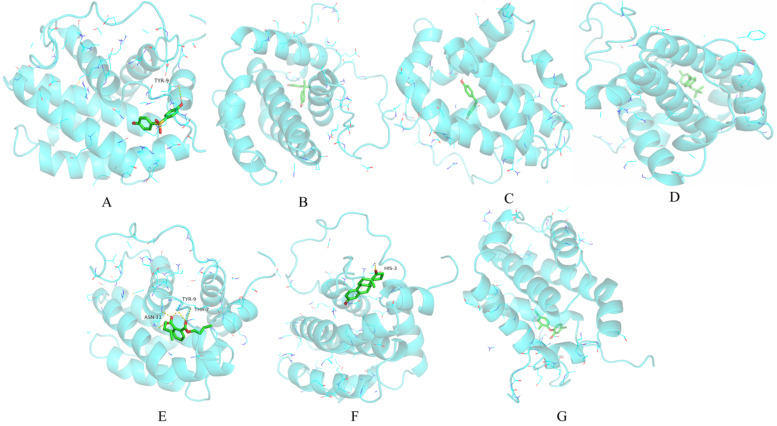
Molecular docking. (A) 4,4 '- Sulfonyldiphenyl docked with BCL2; (B) Bisphenol A docking with BCL2; (C) Bisphenol F docking with BCL2; (D) DDT docking with BCL2; (E) Dibutyl phthalate docking with BCL2; (F) Estrone docking with BCL2; (G) Triclosan docking with BCL2.

Molecular docking was performed between BCL2 and 7 different types of EDCs, and the results showed that these EDCs have strong binding affinity for BCL2 protein. Fig. 6 shows the visualization of the lowest binding energy complexes formed between 7 EDCs and BCL2 proteins. The molecular docking validation results are shown in Table 4.

**Table 4:** Verification results of molecular docking.

targets	Ingredients	Affifinity (kcal/mol)	Distance from best mode	
			rmsd l.b.	rmsd u.b.
BCL2	4,4 '- Sulfonyldiphenyl	−6.4	0	0
	Bisphenol A	−5.3	0	0
	Bisphenol F	−4.4	0	0
	DDT	−5.5	0	0
	Dibutyl phthalate	−5.3	0	0
	Estrone	−7.2	0	0
	Triclosan	−5.4	0	0

### 3.4 Effects of EDCs on type 2 diabetes

**Fig. 7:**
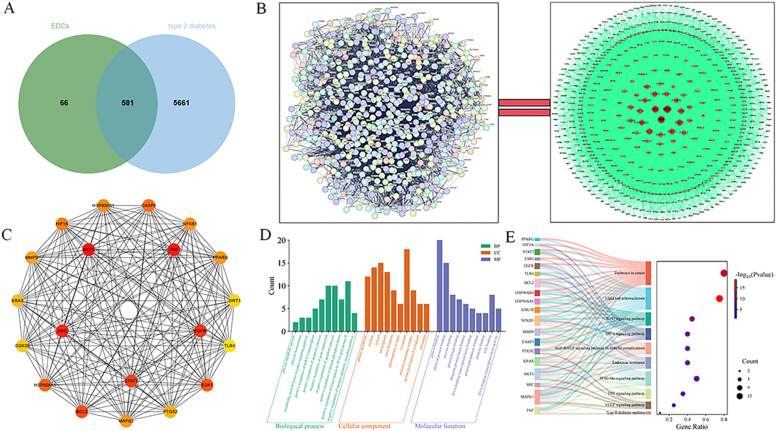
The associations between EDCs and type 2 diabetes. (A) The Venn diagram of related targets between type 2 diabetes and EDCs. (B) PPI network diagram of core targets. (C) Top 20 Hubba gene of core targets.(D) GO enrichment results. (E) The top 10 KEGG enrichment results.

We retrieved 6242 type 2 diabetes mellitus-related genes from the GeneCards, OMIM, and TTD databases along with 647 EDCs-associated genes to generate a Venn diagram (Fig. 7A). This diagram delineates the relationship between EDCs targets and type 2 diabetes mellitus-related targets, identifying 581 overlapping targets that exhibit specific associations with EDCs-induced type 2 diabetes mellitus.

We constructed a PPI network for these 581 overlapping targets using the STRING database and imported the results into Cytoscape software for further analysis (Fig. 7B). In Cytoscape, the Degree algorithm of the CytoHubba plugin was employed to classify disease targets associated with type 2 diabetes mellitus, identifying the top 20 hub genes (Fig. 7C).

Subsequently, GO and KEGG enrichment analyses of these 20 core genes were performed using the DAVID database, with the species restricted to “Homo sapiens”. A total of 132 KEGG terms, along with 250 biological processes (BP), 30 cellular components (CC), and 51 molecular functions (MF), were generated. For GO enrichment results, the top 10 entries in BP, CC, and MF categories were highlighted, respectively, and visualized in a GO enrichment plot (Fig. 7D). For the KEGG enrichment analysis, the top 10 pathways were selected and plotted (Fig. 7E). The results revealed that BP categories were predominantly enriched in glucose metabolic process, insulin-like growth factor receptor signaling pathway, and positive regulation of transcription by RNA polymerase II; CC categories mainly involved nucleus, plasma membrane, and cytoplasm; while MF categories focused on protein binding, ATP binding, and enzyme binding. KEGG pathway analysis demonstrated that EDCs-induced type 2 diabetes mellitus was associated with Lipid and atherosclerosis, Pathways in cancer, and AGE-RAGE signaling pathway in diabetic complications. These findings provide valuable insights into the molecular mechanisms underlying EDCs-induced type 2 diabetes mellitus.

**Fig. 8:**
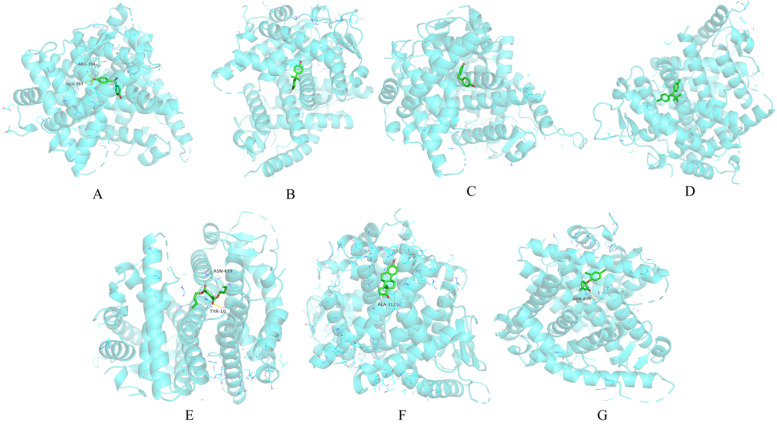
Molecular docking. (A) 4,4 '- Sulfonyldiphenyl docked with ESR1; (B) Bisphenol A docking with ESR1; (C) Bisphenol F docking with ESR1; (D) DDT docking with ESR1; (E) Dibutyl phthalate docking with ESR1; (F) Estrone docking with ESR1; (G) Triclosan docking with ESR1.

Molecular docking was performed between ESR1 and seven distinct types of EDCs, revealing strong binding affinities of these EDCs toward the ESR1 protein. Fig. 8 visualizes the lowest binding energy complexes formed between the seven EDCs and the ESR1 protein. Validation results of molecular docking are presented in Table 5.

**Table 5:** Verification results of molecular docking.

targets	Ingredients	Affifinity (kcal/mol)	Distance from best mode	
			rmsd l.b.	rmsd u.b.
ESR1	4,4 '- Sulfonyldiphenyl	−7.9	0	0
	Bisphenol A	−5.7	0	0
	Bisphenol F	−5.6	0	0
	DDT	−5.8	0	0
	Dibutyl phthalate	−4.9	0	0
	Estrone	−6.7	0	0
	Triclosan	−5.2	0	0

### 3.5 The impact of EDCs on osteoporosis

**Fig. 9:**
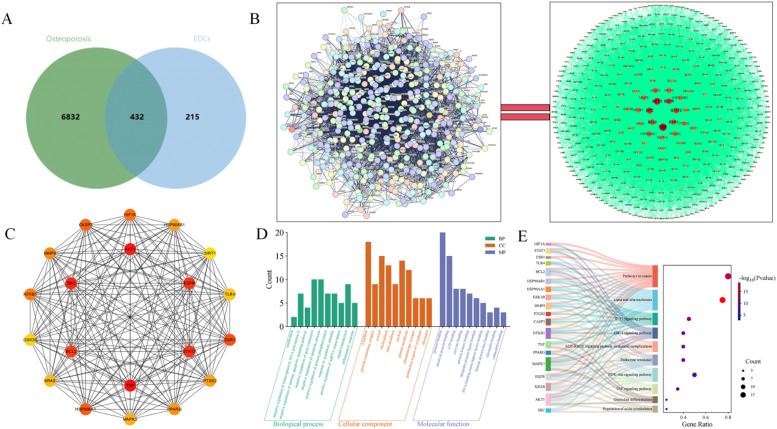
The associations between EDCs and osteoporosis. (A) The Venn diagram of related targets between osteoporosis and EDCs. (B) PPI network diagram of core targets. (C) Top 20 Hubba gene of core targets.(D) GO enrichment results. (E) The top 10 KEGG enrichment results.

We retrieved 7264 osteoporosis-related genes from the GeneCards, OMIM, and TTD databases, and 647 EDCs-related genes to construct a Venn diagram (Fig. 9A). This diagram revealed the relationship between EDCs targets and osteoporosis-related targets, identifying 432 overlapping genes specifically associated with EDCs-induced osteoporosis.

Using the STRING database, we established a PPI network for these 432 overlapping targets and imported the results into Cytoscape for further analysis (Fig. 9B). The CytoHubba plugin in Cytoscape was employed with the Degree algorithm to prioritize disease-related targets, identifying the top 20 core genes (Fig. 9C).

DAVID database was subsequently utilized for GO and KEGG enrichment analyses of these 20 core genes, with species restricted to “Homo sapiens”. This yielded 132 KEGG entries along with 250 biological processes (BP), 30 cellular components (CC), and 51 molecular functions (MF). We highlighted the top 10 entries in each GO category (BP, CC, MF) and generated corresponding enrichment plots (Fig. 9D). For KEGG analysis, the top 10 pathways were visualized (Fig. 9E). The BP category primarily involved signal transduction, negative regulation of apoptotic process, and positive regulation of transcription by RNA polymerase II. CC terms were mainly associated with nucleus, plasma membrane, and cytoplasm. MF categories predominantly included protein binding, ATP binding, and enzyme binding . KEGG pathway analysis revealed significant associations with osteoclast differentiation, cancer pathways, and lipid metabolism/atherosclerosis. These findings provide valuable insights into the molecular mechanisms underlying EDCs-induced osteoporosis.

**Fig. 10:**
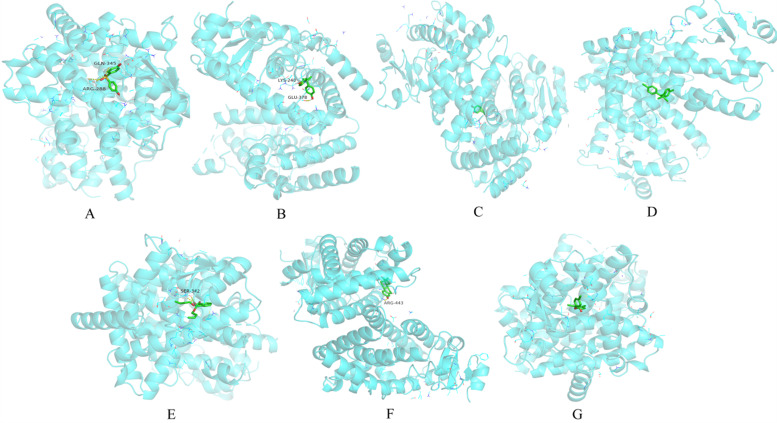
Molecular docking. (A) 4,4 '- Sulfonyldiphenyl docked with PPARG; (B) Bisphenol A docking with PPARG; (C) Bisphenol F docking with PPARG; (D) DDT docking with PPARG; (E) Dibutyl phthalate docking with PPARG; (F) Estrone docking with PPARG; (G) Triclosan docking with PPARG.

Molecular docking was performed between PPARG and seven distinct EDCs types, demonstrating strong binding affinities of these EDCs to the PPARG protein. Fig. 10 visualizes the lowest binding energy complexes formed between the seven EDCs and PPARG. The molecular docking validation results are presented in Table 6.

**Table 6:** Verification results of molecular docking.

targets	Ingredients	Affifinity (kcal/mol)	Distance from best mode	
			rmsd l.b.	rmsd u.b.
PPARG	4,4 '- Sulfonyldiphenyl	−7.0	0	0
	Bisphenol A	−5.7	0	0
	Bisphenol F	−5.5	0	0
	DDT	−6.1	0	0
	Dibutyl phthalate	−6.6	0	0
	Estrone	−7.3	0	0
	Triclosan	−6.8	0	0

### 3.6 The effect of EDCs on hyperuricemia

**Fig. 11:**
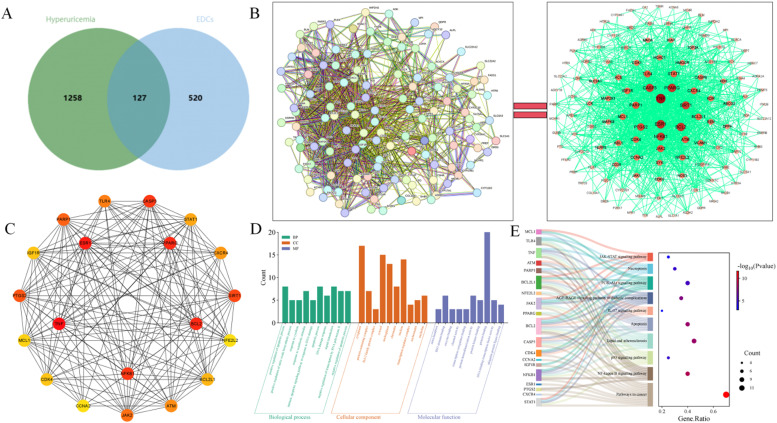
The associations between EDCs and hyperuricemia. (A) The Venn diagram of related targets between hyperuricemia and EDCs. (B) PPI network diagram of core targets. (C) Top 20 Hubba gene of core targets.(D) GO enrichment results. (E) The top 10 KEGG enrichment results.

We obtained 1385 hyperuricemia-related genes from the GeneCards, OMIM, and TTD databases, along with 647 EDCs-related genes, to construct a Venn diagram (Fig. 11A). This visualization revealed the relationship between EDCs targets and hyperuricemia-associated targets, identifying 127 overlapping genes specifically linked to EDCs-induced hyperuricemia.

Using the STRING database, we constructed a PPI network for these 127 overlapping targets and imported the results into Cytoscape for subsequent analysis (Fig. 11B). The CytoHubba plugin in Cytoscape was employed with the Degree algorithm to prioritize disease-related targets, ultimately identifying the top 20 core genes (Fig. 11C).

GO and KEGG enrichment analyses of these 20 core genes were performed using the DAVID database, with species specification set to “Homo sapiens”. This analysis yielded 79 KEGG entries along with 192 biological processes (BP), 24 cellular components (CC), and 37 molecular functions (MF). The top 10 entries from each GO category (BP, CC, MF) were visualized in corresponding enrichment plots (Fig. 11D). For KEGG analysis, the top 10 pathways were graphically represented (Fig. 11E). The BP category primarily involved response to xenobiotic stimulus, apoptotic process, and positive regulation of transcription by RNA polymerase II. CC terms showed predominant associations with nucleus, nucleoplasm, and cytoplasm. MF categories were mainly enriched in protein binding, ATP binding, and enzyme binding. KEGG pathway analysis revealed significant associations with NF-kappa B signaling pathway, cancer pathways, and lipid metabolism/atherosclerosis. These findings provide critical insights into the molecular mechanisms underlying EDCs-induced hyperuricemia.

**Fig. 12:**
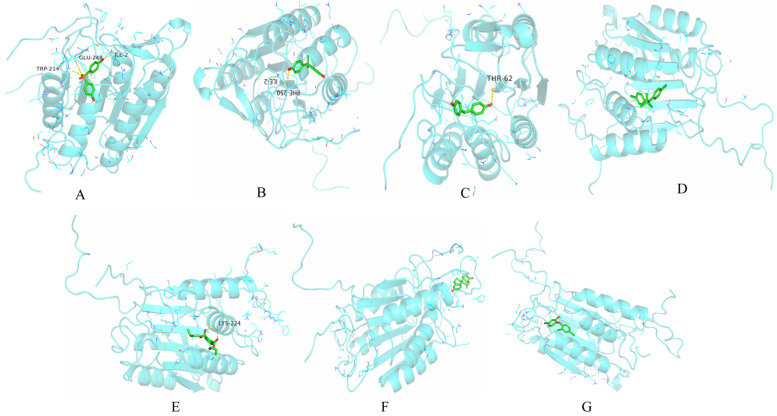
Molecular docking. (A) 4,4 '- Sulfonyldiphenyl docked with CASP3; (B) Bisphenol A docking with CASP3; (C) Bisphenol F docking with CASP3; (D) DDT docking with CASP3; (E) Dibutyl phthalate docking with CASP3; (F) Estrone docking with CASP3; (G) Triclosan docking with CASP3.

Molecular docking analysis between CASP3 and seven distinct EDCs types demonstrated strong binding affinities of these compounds to the CASP3 protein. Fig. 12 illustrates the lowest binding energy complexes formed between the seven EDCs and CASP3. Validation results of the molecular docking experiments are presented in Table 7.

**Table 7:** Verification results of molecular docking.

targets	Ingredients	Affifinity (kcal/mol)	Distance from best mode	
			rmsd l.b.	rmsd u.b.
CASP3	4,4 '- Sulfonyldiphenyl	−6.3	0	0
	Bisphenol A	−4.9	0	0
	Bisphenol F	−5.4	0	0
	DDT	−5.3	0	0
	Dibutyl phthalate	−4.8	0	0
	Estrone	−6.4	0	0
	Triclosan	−5.5	0	0

### 3.7 The effect of EDCs on non-alcoholic fatty liver disease

**Fig. 13:**
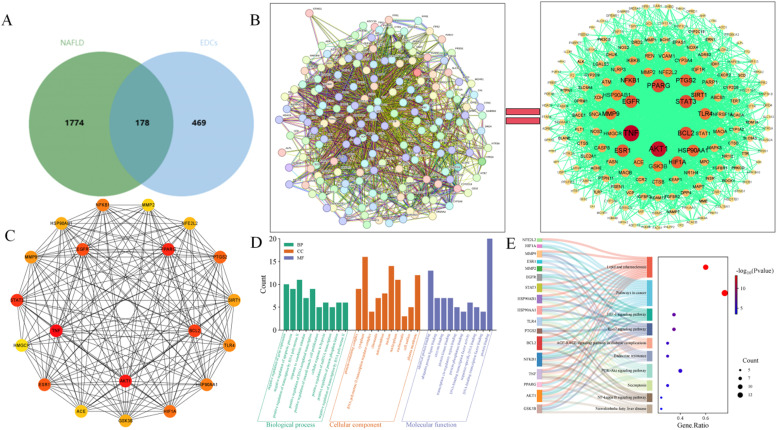
The associations between EDCs and NAFLD. (A) The Venn diagram of related targets between NAFLD and EDCs. (B) PPI network diagram of core targets. (C) Top 20 Hubba gene of core targets.(D) GO enrichment results. (E) The top 10 KEGG enrichment results.

We retrieved 1952 non-alcoholic fatty liver disease (NAFLD)-related genes from the GeneCards, OMIM, and TTD databases, along with 647 EDCs-related genes, to construct a Venn diagram (Fig. 13A). This diagram delineated the relationship between EDCs targets and NAFLD-associated targets, identifying 178 overlapping genes specifically associated with EDCs-induced NAFLD.

A PPI network was established for these 178 overlapping targets using the STRING database, and the results were imported into Cytoscape for further analysis (Fig. 13B). The CytoHubba plugin in Cytoscape was employed with the Degree algorithm to prioritize disease-related targets, ultimately identifying the top 20 core genes (Fig. 13C).

GO and KEGG enrichment analyses of these 20 core genes were performed using the DAVID database, with species restriction set to Homo sapiens. The analysis yielded 96 KEGG entries along with 223 biological processes (BP), 32 cellular components (CC), and 49 molecular functions (MF). The top 10 entries from each GO category (BP, CC, MF) were visualized in corresponding enrichment plots (Fig. 13D). For KEGG analysis, the top 10 pathways were graphically represented (Fig. 13E). The BP category primarily involved negative regulation of apoptotic process, cellular response to hypoxia, and positive regulation of transcription by RNA polymerase II. CC terms showed predominant associations with nucleus, nucleoplasm, and cytoplasm. MF categories were mainly enriched in protein binding, ubiquitin protein ligase binding, and enzyme binding. KEGG pathway analysis revealed significant associations with PI3K-Akt signaling pathway, cancer pathways, and lipid metabolism/atherosclerosis. These findings provide mechanistic insights into the molecular basis of EDCs-induced NAFLD.

**Fig. 14:**
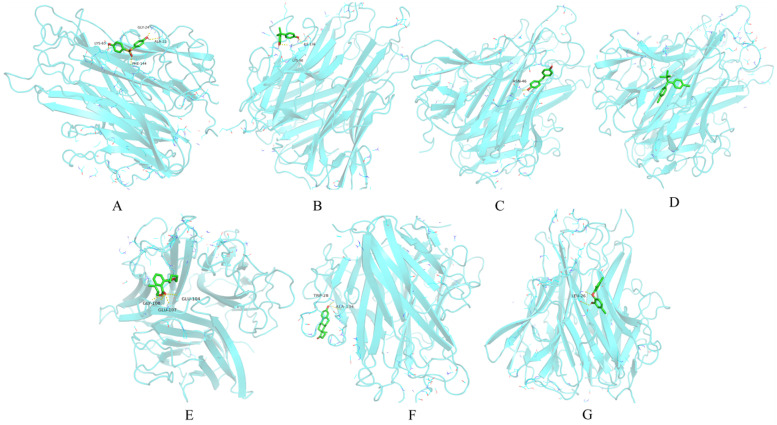
Molecular docking. (A) 4,4 '- Sulfonyldiphenyl docked with TNF; (B) Bisphenol A docking with TNF; (C) Bisphenol F docking with TNF; (D) DDT docking with TNF; (E) Dibutyl phthalate docking with TNF; (F) Estrone docking with TNF; (G) Triclosan docking with TNF.

Molecular docking analysis between TNF and seven distinct EDCs types demonstrated strong binding affinities of these compounds to the TNF protein. Fig. 14 illustrates the lowest binding energy complexes formed between the seven EDCs and TNF. Validation results of the molecular docking experiments are presented in Table 8.

**Table 8:** Verification results of molecular docking.

targets	Ingredients	Affifinity (kcal/mol)	Distance from best mode	
			rmsd l.b.	rmsd u.b.
TNF	4,4 '- Sulfonyldiphenyl	−5.5	0	0
	Bisphenol A	−4.7	0	0
	Bisphenol F	−5.1	0	0
	DDT	−5.3	0	0
	Dibutyl phthalate	−3.7	0	0
	Estrone	−6.5	0	0
	Triclosan	−5.4	0	0

## 4 Discussion

We utilized a comprehensive suite of databases (including PubChem, ChEMBL, Super-PRED, GeneCards, OMIM, TTD, and STRING) to retrieve and analyze relevant targets. Subsequently, Cytoscape software was employed to conduct a systematic analysis of these targets to identify core targets. Gene Ontology (GO) and Kyoto Encyclopedia of Genes and Genomes (KEGG) enrichment analyses were then performed on the core targets using the DAVID database. Finally, molecular docking studies were carried out with PyMOL and AutoDock Vina, enabling us to explore the intermolecular interactions between EDCs and core target proteins. Collectively, our findings elucidate the potential molecular mechanisms by which EDCs induce metabolic diseases in humans.

According to the World Health Organization (WHO), environmental factors account for 22 % of the global disease burden and 23 % of deaths worldwide (Najera and Reiss, 2016). Among these environmental hazards, EDCs are predominant (Hang et al., 2021). In China, anthropogenic EDCs, including organochlorine pesticides, DDT, and BPA, are widely detected across various environmental media (Shanlin and Jianbo, 2005). With societal advancements and lifestyle changes, EDCs may pose a significant threat to human health in the near future if left unregulated. Furthermore, EDCs have been shown to bioaccumulate in organisms, severely disrupting lipid metabolism in fish (Shengxiang and Zhenyu, 2019), inducing gonadal malformations during development (Bernet et al., 2008), impairing reproductive capacity (Milestone et al., 2011), and even triggering sex reversal from male to hermaphroditic or female phenotypes (Kidd et al., 2007; Baroiller and Guiguen, 2001). Studies have shown that EDCs can cause immune system damage (Sabuz Vidal et al., 2021), cancer (Modica et al., 2023), cardiovascular risk (Xiaolian et al., 2023), behavioral disorders (You et al., 2023) and reproductive diseases (Thacharodi et al., 2023; Ghosh et al., 2022).To advance our understanding of EDCs' impact on human health. The OBERON project mentions that endocrine disruptors are one of the most serious public health threats today, and establishes a comprehensive testing strategy to detect metabolic disorders related to endocrine disruptors through the development, improvement, and validation of a series of testing systems (XXXX, 2018). In addition, recent evidence from the EDCMET project shows that the increase in the incidence rate of metabolic syndrome is related to EDCs, thus increasing the incidence rate of atherosclerosis and type 2 diabetes (Kublbeck et al., 2020; XXXX, 2024). this study employs network toxicology and molecular docking techniques to investigate the molecular mechanisms underlying EDCs-induced metabolic disorders.

The analysis of Gene Ontology (GO) and Kyoto Encyclopedia of Genes and Genomes (KEGG) pathways related to lipids and atherosclerosis revealed that the PI3K-Akt signaling pathway and the SRC gene are key pathways and critical genes involved. The PI3K-Akt signaling pathway, as a major intracellular signaling route, plays an essential role in various biological processes such as cellular metabolism, cell cycle regulation, cell proliferation, and apoptosis. It is implicated in all stages of atherosclerosis pathogenesis (Wanshou and Hua, 2020; Zhenzhu et al., 2022). Furthermore, SRC may regulate the formation of atherosclerotic plaques by mediating lipid accumulation in macrophages (Qing et al., 2016). These findings align with our current research results and further validate our conclusions. Additionally, diabetes has been shown to promote the development of atherosclerosis primarily through transcriptional changes in vascular cells associated with turbulent blood flow, inflammation, and oxidative stress (Khan and Jandeleit-Dahm, 2025).

Regarding Alzheimer's disease (AD), our study indicates that its onset may be linked to neuronal apoptosis processes involving the BCL2 gene. In patients with AD, necrotic apoptosis occurs within brain cells leading to neuronal death; this contributes to disease progression characterized by cognitive decline and brain tissue atrophy (Caccamo et al., 2017). Members of the Bcl-2 protein family play crucial roles in maintaining cellular health; Bcl-2 specifically acts on mitochondria to control apoptotic initiation while participating in intracellular Ca²⁺ signal transduction—key processes for neuronal function (Callens et al., 2021). Dysregulation of Bcl-2 can lead to the onset and progression of AD. Moreover, upon receiving pro-apoptotic signals, members of the Bcl-2 family form an intricately complex interaction network regulating β-cell apoptosis which ultimately leads to type 2 diabetes mellitus (T2DM) (Hong and Dong, 2011). Notably, Bcl-2 ranks among the top 20 hub genes associated with T2DM. In our investigation into T2DM mechanisms, we selected ESR1 for molecular docking studies alongside endocrine-disrupting chemicals (EDCs), revealing strong binding affinity between them. This suggests that ESR1 could serve as a potential inducer factor for T2DM development. One study indicated that activation of ESR1 within adipocytes increases nuclear content levels of SP1 protein along with enhanced interactions between SP1/ESR1 as well *As sp1*'s binding capacity at Slc2a4 gene promoters—ultimately resulting in increased expression levels of Slc2a4/GLUT4 which promotes insulin resistance contributing to diabetes progression (Barreto-Andrade et al., 2018). This further corroborates our research findings.

In osteoporosis, we have found that its occurrence is related to the differentiation of osteoclasts. Previous studies have indicated that osteoclast differentiation plays a crucial role in the pathogenesis of osteoporosis (Xiangfu et al., 2015). One study revealed that in an ovariectomized (OVX) mouse model, TET2 downregulates the expression of BCL2 and enhances BECN1-dependent autophagy, thereby promoting osteoclast activation and bone mass loss, which contributes to the development and progression of osteoporosis (Yang et al., 2022). Interestingly, the BCL2 gene also plays a significant role in this process. Additionally, PPARG nuclear receptors can regulate energy metabolism and insulin sensitivity; furthermore and the transcriptional activity of PPARG is essential for the expression of sclerotic proteins in bone cells (Baroi et al., 2021). This finding corroborates our research.

Regarding hyperuricemia, we selected CASP3 for molecular docking with EDCs, revealing strong binding affinity. Currently, there is no direct evidence linking CASP3 with hyperuricemia; however, hyperuricemia increases reactive oxygen species (ROS) production, which may activate CASP3 through mitochondrial apoptotic pathways (Liu et al., 2021). Conversely, activation of CASP3 could further enhance oxidative stress, creating a vicious cycle that promotes disease progression (Linchun et al., 2022). Moreover, multiple studies suggest that CASP3 represents a potential therapeutic target for hyperuricemia treatment (Xiaoni et al., 2021; Siting et al., 2024). These findings indirectly reflect a close relationship between CASP3 and hyperuricemia consistent with our research outcomes. Furthermore, CASP3 is closely associated with hepatocyte apoptosis in NAFLD and participates in its pathogenesis (Haifeng et al., 2012). It is noteworthy that CASP3 is also one of the core genes involved in NAFLD. For NAFLD analysis, we conducted molecular docking between TNF and EDCs and discovered their strong binding capacity—suggesting TNF may be an important factor contributing to NAFLD development (Vachliotis and Polyzos, 2023). TNF-α is a cytokine playing a critical role in the pathogenesis of NAFLD; although its production might occur as an early event during NAFLD progression it could potentially exacerbate hepatic insulin resistance by linking inflammation with metabolic signaling pathways—thereby leading to complications associated with NAFLD. One study indicates that TNF-α significantly influences both the onset and progression of NAFLD by inducing insulin resistance while contributing to metabolic syndrome formation among patients suffering from this condition (Lingli et al., 2010). Therefore, there exists a high degree of correlation among these six metabolic diseases.

In our study, we identified 7 common genes among the top 20 hub genes associated with the aforementioned 6 diseases: BCL2, PTGS2, TLR4, PPARG, TNF, ESR1, and NFKB1. Therefore, we hypothesize that patients with one of these diseases may exhibit changes in the expression levels of the aforementioned genes compared to healthy individuals. This differential gene expression may promote the development of another disease. Our research findings further indicate a significant correlation between these diseases. In addition, the selected core targets can provide new insights for the development and innovation of targeted therapeutic drugs for treating diseases; And it can further promote the development of drugs aimed at preventing and treating other comorbidities or related diseases; In addition, the understanding of the complex mechanisms by which different pollutants cause diseases has been strengthened, which will greatly enhance public health and disease prevention strategies.

## 5 Conclusion

In summary, we have identified numerous potential targets of EDCs associated with metabolic diseases using network toxicology and molecular docking techniques. These findings elucidate the potential mechanistic connections between EDCs and six common metabolic diseases. This study provides valuable insights for the fields of toxicology and environmental health, and offers innovative approaches for exploring the impact of a range of pollutants on human diseases. Based on these findings, we will further explore how environmental factors affect the incidence rate and progress of various human diseases. Ultimately, we hope that this research can resonate with people around the world to take collective action to mitigate the impact of EDCs on ecosystems and public health.

## Abbreviations

**Table:** 

EDCs	Endocrine-disrupting Chemicals
KEGG	Kyoto Encyclopedia of Genes and Genomes
GO	Gene Ontology
DEHP	Di-2-ethylhexyl phthalate
PFAS	Polyfluoroalkyl Substances
NAFLD	Non-alcoholic Fatty Liver Disease
T2DM	Type 2 Diabetes Mellitus
PPARγ	Peroxisome Proliferator-activated Receptor gamma
AD	Alzheimer's disease
STAT3	Signal transducer and activator of transcription 3
SREBP-1c	Sterol regulatory element-binding protein 1c
SRC	Non receptor tyrosine kinase
PI3K-Akt	Phosphatidylinositol 3 kinase/protein kinase B
BCL2	B-cell lymphoma-2
MEG3	Maternally Expressed 3
ESR1	Estrogen Receptor alpha
SP1	Specific protein 1
Slc2a4	Solute Carrier Family 2 Member 4
GLUT4	Glucose Transporter 4
TET2	Ten-eleven Translocation 2
BECN1	autophagy related Gene 1
PPARG	Peroxisome Proliferator-activated Receptor Gamma
TNF-α	Tumor Necrosis Factor α
PTGS2	Prostaglandin-endoperoxide Synthase 2
TLR4	Toll-like receptor 4
NFKB1	nuclear factor kappa-B1
LPCAT3	lysophosphatidylcholine acyltransferase 3

## Ethics declarations

Not Applicable

## CRediT authorship contribution statement

**Min Zhao:** Writing – original draft, Data curation, Conceptualization. **Yong Niu:** Software, Data curation. **Qian Huang:** Validation, Software. **Wenhua Li:** Writing – review & editing.

## Declaration of competing interest

The authors declare that they have no known competing financial interests or personal relationships that could have appeared to influence the work reported in this paper.

## Data Availability

Data will be made available on request.
